# Network structure of common mental health problems and life satisfaction in a Japanese population

**DOI:** 10.1038/s41598-025-95554-1

**Published:** 2025-04-10

**Authors:** Shota Noda, Motohiro Nishiuchi, Maaya Kobayashi, Stefan G. Hofmann

**Affiliations:** 1 Department of Psychology, Philipps-University of Marburg, Schulstraße 12, 35032 Marburg, Germany; 2 Research Institute of Cognitive Behavior Therapy, Musashino University, 3-3-3 Ariake, Koto-ku, Tokyo 135-8181 Japan; 3 Graduate School of Human and Social Sciences, Musashino University, 3-3-3 Ariake, Koto-ku, Tokyo 135-8181 Japan

**Keywords:** Network analysis, Mental health, Anxiety, Depression, Loneliness, Satisfaction with life, Psychology, Health care

## Abstract

Poor life-satisfaction, feelings of loneliness, depression, and anxiety are commonly reported in the Japanese general population. The aim of this study was to examine the network structure of these common mental health problems in a Japanese sample and to explore differences between males and females. Participants (*N* = 476; 235 males, 241 females) were recruited from a Japanese general population and completed questionnaires to measure levels of depression, anxiety, loneliness, and life satisfaction. A psychometric network analysis was conducted using a Gaussian Graphical Model, and a network comparison test (NCT) was performed to evaluate overall consistency and differences in global network connectivity. The network analysis identified problems relaxing, sad mood, feelings of isolation, and poor life satisfaction as central nodes in the overall network. The NCT revealed no significant differences between the male and female networks in the global network structure as well as connectivity. However, the strength values for restlessness and tiredness were significantly higher in the network for males than in the network for females. There were no differences in the global network structure and connectivity of mental health psychopathology between males and females. Problems relaxing, feelings of depression, being isolated from others, and poor life satisfaction contribute to poor mental health and may be potential intervention targets.

**Supplementary Information:**

The online version contains supplementary material available at 10.1038/s41598-025-95554-1.

## Introduction

Mental health problems are common in Japanese population. The World Mental Health Japan 2nd Survey, conducted between 2013 and 2015, estimated the lifetime prevalence of major depressive disorder at 5.7% and anxiety disorders at 4.2%^1^. According to the Ministry of Health, Labour and Welfare of Japan^2^, over 82% of individuals report experiencing work-related mental health problems, including those associated with job responsibilities, workload, and interpersonal relationships. The number of suicides in 2023 was 21,837^3^, which was relatively high compared to other countries. Despite the importance of promoting mental health, mental health care in Japan remains inadequate, particularly in the field of primary prevention^2^.

Mental health can be defined as a state of mental well-being that enables people to cope with the stresses of life, realize their abilities, learn well and work well, and contribute to their community^4^. In other words, it encompasses fewer symptoms of anxiety and depression, positive and well-functioning community relationships, and high levels of life satisfaction. Loneliness, recognized as one of public mental health problems, is linked to the subsequent onset of other mental health problems, such as anxiety and depression, as well as lower life satisfaction^5–7^. Recent studies indicate that a considerable number of individuals experience some degree of loneliness (e.g.,^8^). Indeed, 4.8% of Japanese individuals reported always or often feeling lonely, while 14.8% reported sometimes feeling lonely^9^. These findings highlight the importance of addressing loneliness as a key factor in the broader context of mental health.

To promote mental health care, it is important to understand the nature of psychopathology and mental health. In recent years, psychometric network analysis has emerged as a valuable tool for examining the complexities of psychopathology^10^. This approach validates the interconnections among variables within a network and identifies key elements that play pivotal roles in the network’s maintenance and exacerbation^11,12^. In network analysis, nodes represent observable variables, while edges denote the relationships between these nodes. Central nodes—symptoms that are critical to the maintenance and exacerbation of the network—are considered priority targets for intervention^11^.

Previous studies have highlighted the centrality of various symptoms in mental health networks. Beard et al.^13^ investigated the network structure of anxiety and depression in an American psychiatric population and identified “sad mood” as a central symptom. Similarly, Cai et al.^14^ examined the network structure of anxiety and depression among Chinese adolescents and also identified “sad mood” as central. Ochnik et al.^15^ explored the network structure of anxiety, depression, life satisfaction, physical health, and perceived stress in a Czech student sample and also identified “sad mood” as a central symptom. Ma et al.^16^ reported that “lack of happiness” showed the strongest negative correlation with life satisfaction in the anxiety and depression network among older Chinese adults with hypertension. Yang et al.^17^ analyzed the network structure of loneliness and symptoms of anxiety and depression in a Chinese student sample, identifying “people are around me but not with me” as the central symptom for the loneliness-anxiety network and “anhedonia” for the loneliness-depression network.

Since many Japanese individuals experience loneliness and tend to have poor life satisfaction as well as poor physical and mental health^9^, a complex relationship between anxiety, depression, loneliness, and life satisfaction can be assumed. However, to the best of our knowledge, no studies have examined the mental health network of the Japanese population or explored the centrality of symptoms within a network consisting of such. Therefore, this study aimed to examine the network structure of mental health comprising symptoms of anxiety and depression, loneliness, and life satisfaction to identify central symptoms and gain deeper insights into the psychopathology of mental health in the Japanese population. Given that sex differences have been reported in mental health-related variables such as anxiety, depression, and loneliness^18–20^, we also explored differences in mental health networks between males and females to identify potential variations in psychopathology.

## Methods

### Participants

This study analyzed data collected in March 2024 as part of a prior research project^21^. A total of 500 participants from the general Japanese population were recruited through the online research platform Rakuten Insight. Eligibility criteria required that participants be at least 20 years old and reside in Japan. Before completing the questionnaires, all participants provided informed consent and, upon completion, received Rakuten points redeemable for various Rakuten services. All questionnaire items were mandatory to ensure data completeness, resulting in no missing responses. Response validity was assessed using the Directed Questions Scale (DQS^22^). Participants who failed to respond appropriately to the two designated DQS items were excluded from the analysis. Consequently, 24 individuals were removed from this study, leaving a final sample of 476 participants (mean age = 45.59, SD = 13.55; 235 males, 241 females). Ethical approval for the original study was obtained from the ethics committee of the first author’s affiliated university (Approval No. 2023-33-01).

### Materials

#### Japanese version of the generalized anxiety disorder-7

To measure anxiety symptoms, we used the Japanese version of the Generalized Anxiety Disorder-7 (GAD), developed by Shimizu et al.^23^. This scale includes seven items, each scored on a 4-point scale where 0 indicates “not at all” and 3 indicates “nearly every day.” The GAD demonstrated high reliability in this study, with Cronbach’s α coefficient calculated at 0.90 and McDonald’s ω at 0.91.

#### Japanese version of the patient health questionnaire-9

To assess symptoms of depression, we used the Japanese version of the Patient Health Questionnaire-9 (PHQ), developed by Shimizu et al.^23^. This scale contains nine items, each rated on a 4-point scale ranging from 0 (“not at all”) to 3 (“nearly every day”). The PHQ-9 exhibited high reliability in this study, with Cronbach’s α coefficient calculated at 0.89 and McDonald’s ω at 0.89.

#### Japanese version of the short form of the UCLA loneliness scale

To measure loneliness, we used the short-form Japanese version of the UCLA Loneliness Scale (ULS), as adapted by Igarashi^24^. This scale comprises three items scored on a 3-point scale ranging from 1 (“hardly ever”) to 3 (“often”). The ULS-3 showed high reliability in this study, with Cronbach’s α coefficient calculated at 0.84 and McDonald’s ω at 0.85.

#### Japanese version of the satisfaction with life scale

To assess life satisfaction, we used the Japanese version of the Satisfaction With Life Scale (SWLS), developed by Sumino^25^. This scale consists of five items, each rated on a 7-point scale from 1 (“strongly disagree”) to 7 (“strongly agree”). The SWLS demonstrated high reliability in this study, with Cronbach’s α coefficient calculated at 0.90 and McDonald’s ω at 0.91.

### Statistical analyses

First, the differences in mental health-related variables between males and females were examined using *t*-tests in SPSS version 28.0 (IBM Corp., Armonk, NY, USA). Second, a psychometric network analysis was conducted using R version 4.4.2 (R Core Team, 2024) with the R package qgraph^26^. The analysis employed a Gaussian Graphical Model to estimate the network structure. Edges within the network represent partial correlations after controlling for all other nodes^27^. Blue edges indicate positive correlations, red edges indicate negative correlations, and thicker, brighter lines show stronger connections. The estimation applied graphical least absolute shrinkage and selection operator regularization^28^ and the Extended Bayesian Information Criterion^29^. To evaluate the central symptoms of the network, which are critical to the maintenance and exacerbation of the network, we examine centrality indices. The indicators for centrality indices included strength (the total weight of the edges connected to a node), closeness (the average distance from a node to all other nodes), and betweenness (the frequency with which a node lies on the shortest path between two distinct nodes)^12^. The accuracy of the edge weights was evaluated with bootstrapped 95% confidence intervals (nboots = 2,500) using the R package bootnet^27^. To assess the stability of centrality indices, the correlation stability (CS) coefficient was computed based on 2,500 bootstrap samples. According to Epskamp et al.^27^, the CS (cor = 0.7) coefficient represents the highest percentage of cases that can be removed while maintaining a 95% probability and a correlation of 0.7 between the original centrality indices and those derived from subsets. CS coefficients of 0.50 or higher are desirable, whereas those below 0.25 are not interpretable. Therefore, we interpreted centrality based on CS coefficients greater than 0.25 in this study. Network structures were compared using the R package Network Comparison Test (NCT^30^) to evaluate overall consistency across different groups (i.e., whether connections between nodes were similar) and differences in global network connectivity (i.e., the total sum of absolute edge weights). The NCT also tested specific edge weights and node strengths for group differences.

## Results

### Differences between mental health-related variables between males and females

Table 1 presents the descriptive statistics for each mental health-related variable in males and females, along with the differences between the two groups. Females exhibited significantly higher scores than males in sleep difficulties, appetite, and concentration difficulties related to depressive symptoms (*p* < .05). No significant differences were found between males and females in age, marital status, children, and income (Table S1).

**Table 1:** Node label and descriptive statistics of the participants.

Mental health-related variables	Node label	All participants	Male	Female	Differences between males and females
		Mean(SD)	Mean(SD)	Mean(SD)	*t*-values
Anxiety symptoms					
GAD1	Feeling nervous	0.63(0.84)	0.57(0.78)	0.68(0.89)	− 1.33
GAD2	Uncontrollable worry	0.55(0.82)	0.49(0.81)	0.61(0.84)	− 1.66
GAD3	Worrying too much	0.78(0.90)	0.73(0.90)	0.83(0.89)	− 1.29
GAD4	Trouble relaxing	0.45(0.79)	0.46(0.81)	0.45(0.77)	0.10
GAD5	Restlessness	0.29(0.64)	0.29(0.64)	0.28(0.65)	0.20
GAD6	Irritability	0.64(0.77)	0.59(0.74)	0.69(0.80)	− 1.44
GAD7	Feeling afraid	0.40(0.73)	0.40(0.73)	0.41(0.73)	− 0.10
Depressive symptoms					
PHQ1	Anhedonia	0.71(0.81)	0.70(0.81)	0.71(0.81)	− 0.21
PHQ2	Sad mood	0.66(0.82)	0.61(0.82)	0.70(0.83)	− 1.17
PHQ3	Sleep difficulties	1.06(1.03)	0.96(1.01)	1.16(1.03)	− 2.14**
PHQ4	Tiredness	1.04(0.95)	0.99(0.93)	1.10(0.96)	− 1.25
PHQ5	Appetite	0.63(0.87)	0.46(0.78)	0.80(0.93)	− 4.35**
PHQ6	Worthlessness	0.61(0.94)	0.58(0.94)	0.63(0.95)	− 0.50
PHQ7	Concentration difficulties	0.48(0.82)	0.40(0.75)	0.56(0.87)	− 2.20*
PHQ8	Motor	0.26(0.61)	0.29(0.64)	0.24(0.58)	0.95
PHQ9	Suicidal ideation	0.30(0.68)	0.30(0.66)	0.31(0.71)	− 0.15
Loneliness					
ULS1	Lack of companionship	1.96(0.75)	1.95(0.77)	1.96(0.73)	− 0.14
ULS2	Feeling left out	1.50(0.67)	1.49(0.68)	1.51(0.66)	− 0.34
ULS3	Feeling isolated from others	1.66(0.71)	1.62(0.70)	1.70(0.72)	− 1.23
Satisfaction with life					
SWLS1	Ideal life	3.83(1.62)	3.87(1.64)	3.79(1.61)	0.56
SWLS2	Life in excellent condition	4.02(1.62)	3.96(1.62)	4.07(1.62)	− 0.79
SWLS3	Life satisfaction	4.22(1.68)	4.16(1.68)	4.28(1.68)	− 0.78
SWLS4	Accomplishment	4.18(1.50)	4.17(1.50)	4.19(1.51)	− 0.15
SWLS5	Living the same life if I could live my life over	3.19(1.66)	3.32(1.68)	3.07(1.63)	1.69

*p < 0.05, **p < 0.01.

GAD generalized anxiety disorder-7, *PHQ* patient health questionnaire-9, *SWLS* satisfaction with life scale, *ULS* UCLA loneliness scale.

### Networks structure in total sample

**Fig. 1:**
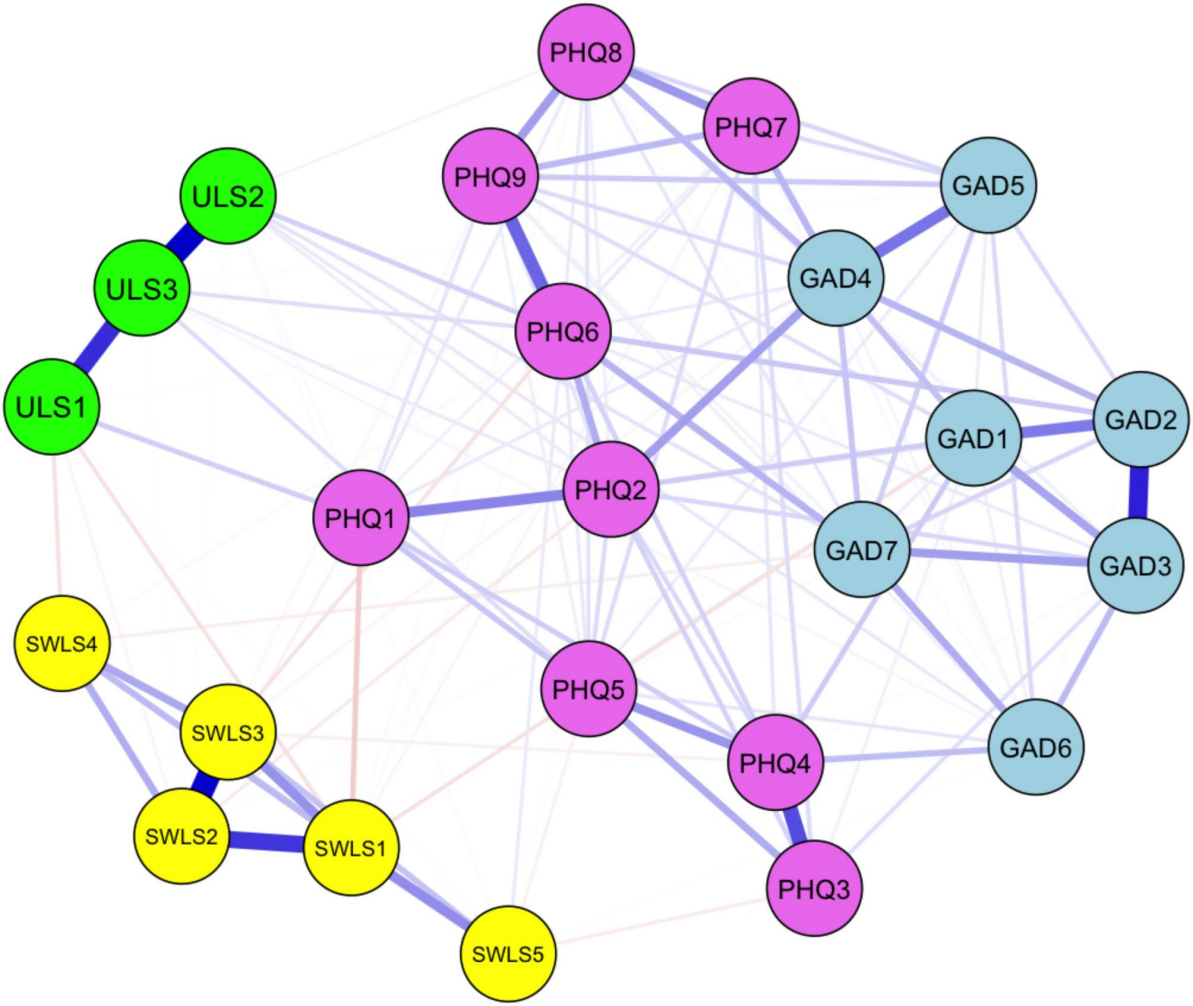
Network structure of depressive and anxiety symptoms, loneliness, and satisfaction with life for all participants. *Note*: GAD = Generalized Anxiety Disorder-7, PHQ = Patient Health Questionnaire-9, SWLS = Satisfaction With Life Scale, ULS = UCLA Loneliness Scale.

**Fig. 2:**
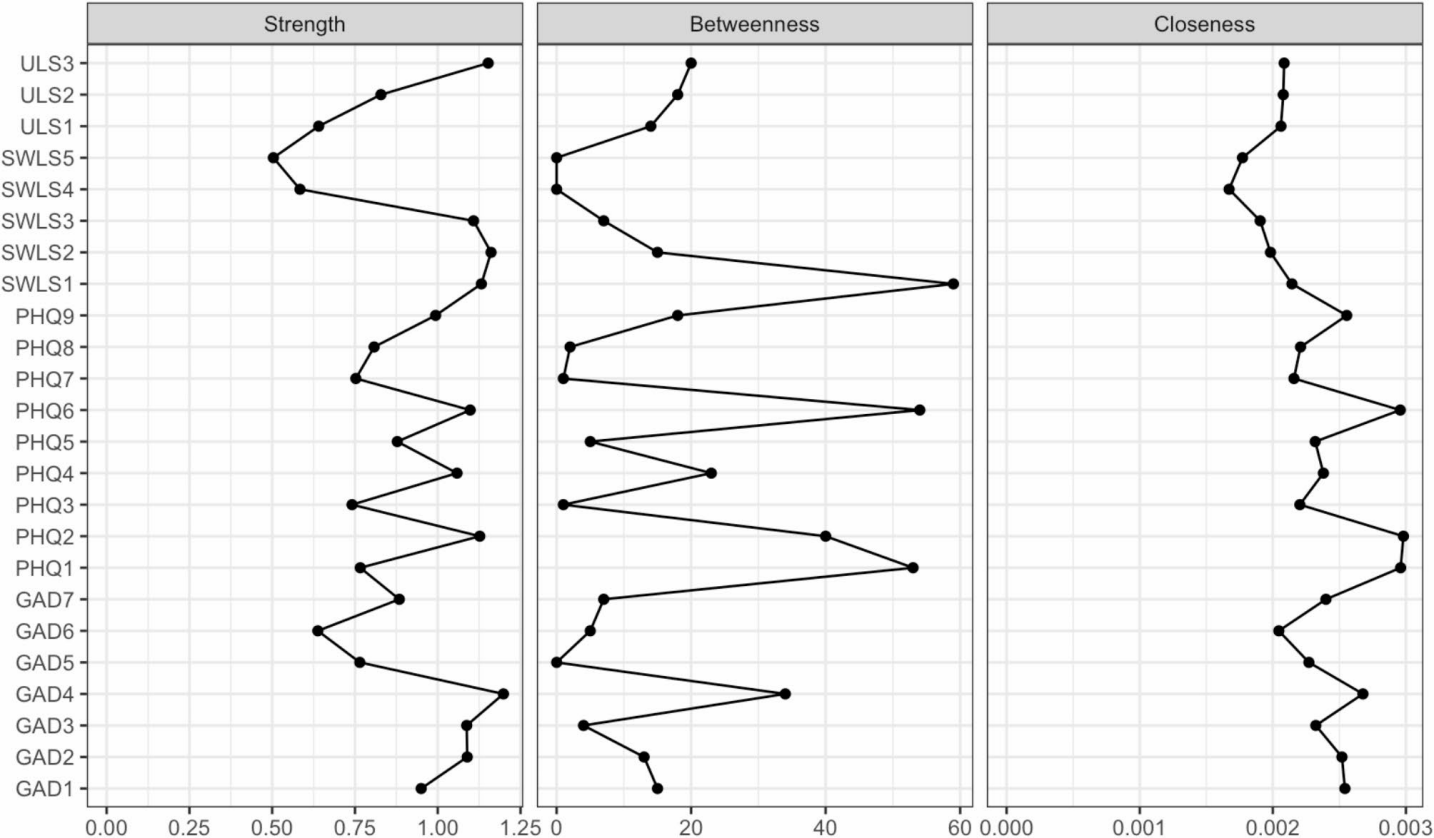
Centrality plot of the networks for all participants (*N* = 476). *Note*: GAD = Generalized Anxiety Disorder-7, PHQ = Patient Health Questionnaire-9, SWLS = Satisfaction With Life Scale, ULS = UCLA Loneliness Scale.

The characteristics of individual nodes are detailed in Table 1, and Fig. 1 illustrates the network structures for all participants. The edge weights are listed in Table S2 (see Figure S1 for the accuracy of the edge weights). The edge weights between anxiety symptoms ranged from 0.000 to 0.432, symptoms of depression from 0.000 to 0.340, loneliness from 0.016 to 0.569, life satisfaction from 0.000 to 0.545, symptoms of anxiety and depression from 0.000 to 0.172, anxiety and loneliness from − 0.005 to 0.049, anxiety and life satisfaction from − 0.051 to 0.000, symptoms of depression and loneliness from 0.000 to 0.086, symptoms of depression and life satisfaction from − 0.089 to 0.035, and loneliness and life satisfaction − 0.053 to 0.000. The CS coefficients for the overall network were recorded as 0.75 for strength, 0.52 for closeness, and 0.21 for betweenness (see Figure S2 for centrality stability). The strength and closeness indices were interpretable. Nodes demonstrating higher strength centrality included “trouble relaxing” (GAD4), “sad mood” (PHQ2), “feeling isolated from others” (ULS3), and “life in excellent condition” (SWLS2) (Fig. 2 and Table S3).

### Sex differences in network structure

**Fig. 3:**
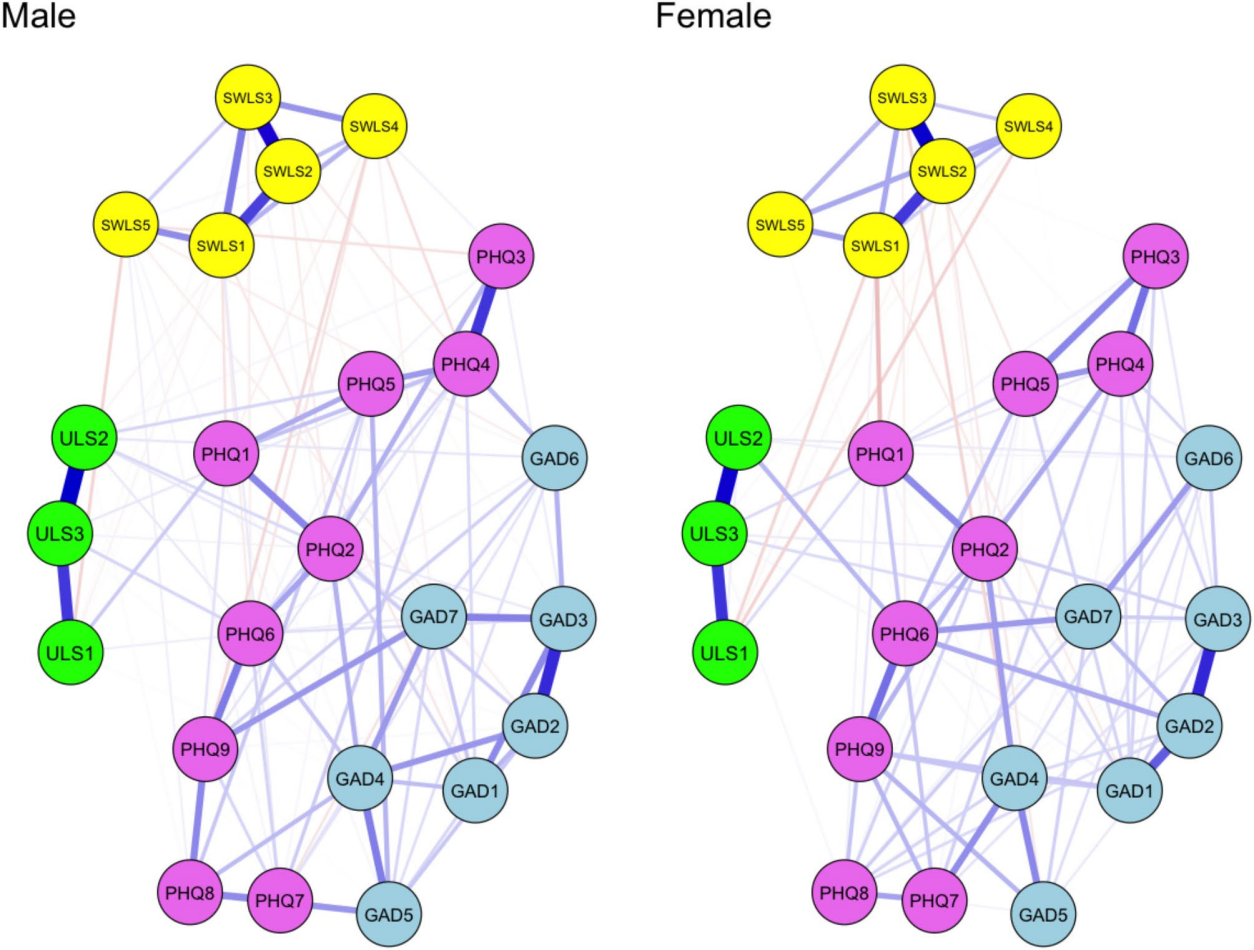
Network structure of depressive and anxiety symptoms, loneliness, and satisfaction with life for males (*n* = 235) and females (*n* = 241). *Note*: GAD = Generalized Anxiety Disorder-7, PHQ = Patient Health Questionnaire-9, SWLS = Satisfaction With Life Scale, ULS = UCLA Loneliness Scale.

The network structures for males and females are illustrated in Fig. 3, while edge weights are provided in Table S4 for the male network and Table S5 for the female networks (see Figure S3 for the accuracy of the edge weights). In the male network, the edge weights between symptoms of anxiety ranged from 0.000 to 0.421, depressive symptoms from 0.000 to 0.396, loneliness from 0.008 to 0.580, life satisfaction from 0.000 to 0.499, symptoms of anxiety and depression from − 0.048 to 0.193, anxiety and loneliness from 0.000 to 0.044, anxiety and life satisfaction from − 0.041 to 0.016, depression and loneliness from 0.000 to 0.091, symptoms of depression and life satisfaction from − 0.069 to 0.028, and loneliness and life satisfaction − 0.075 to 0.000. In the female network, the edge weights between anxiety ranged from 0.000 to 0.428, depression from 0.000 to 0.273, loneliness from 0.030 to 0.534, life satisfaction from 0.000 to 0.549, symptoms of depression and anxiety from 0.000 to 0.210, anxiety and loneliness from 0.000 to 0.063, anxiety and life satisfaction from − 0.076 to 0.003, depression and loneliness from 0.000 to 0.129, symptoms of depression and life satisfaction from − 0.123 to 0.023, and loneliness and life satisfaction − 0.078 to 0.000. The CS coefficients were as follows: 0.60 for strength, 0.36 for closeness, and 0.00 for betweenness in the male network; 0.75 for strength, 0.60 for closeness, and 0.21 for betweenness in the female network (see Figure S4 for centrality stability). The strength and closeness indices were interpretable in both networks.

**Fig. 4:**
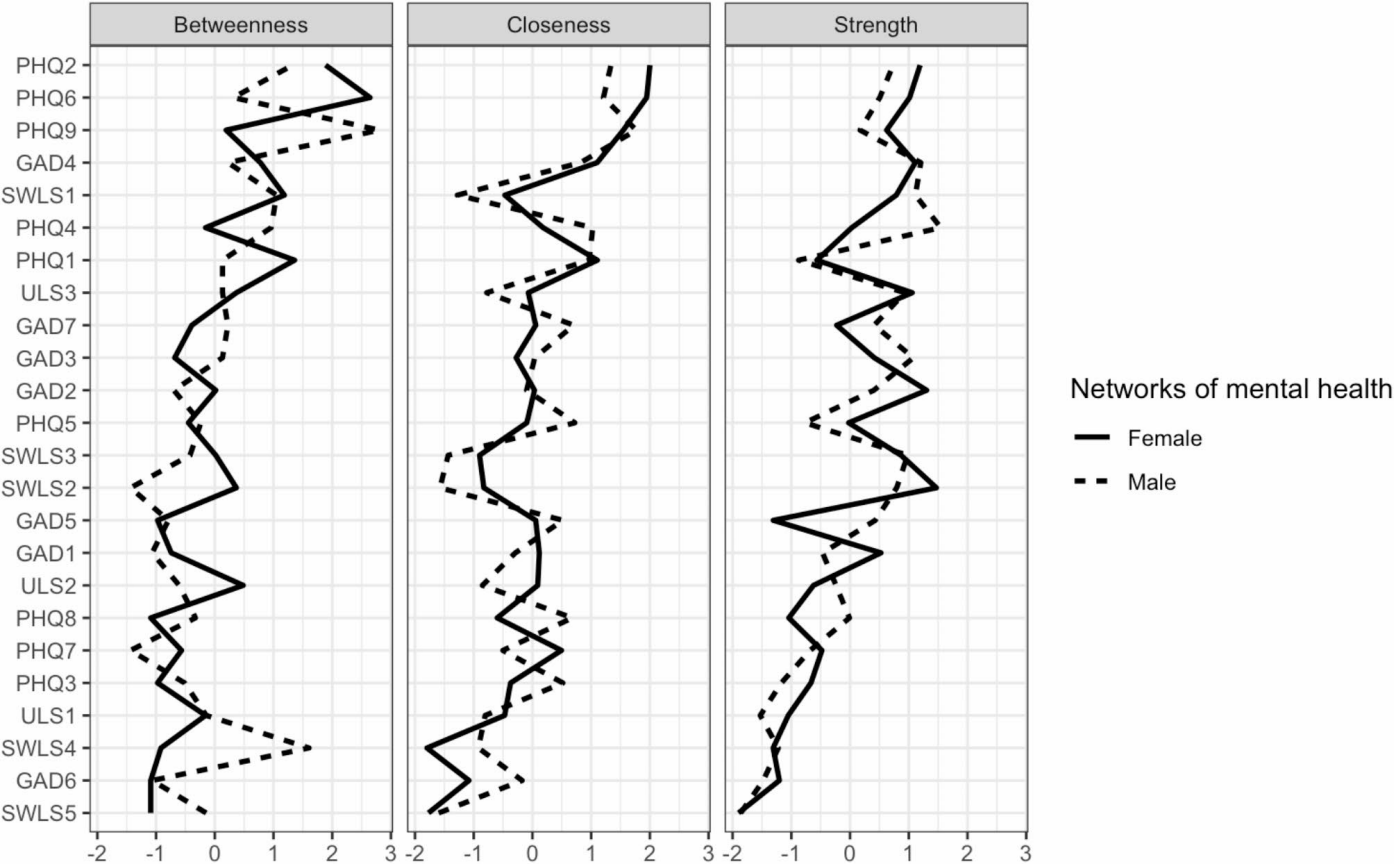
Centrality plot of the networks for males (*n* = 235) and females (*n* = 241). *Note*: GAD = Generalized Anxiety Disorder-7, PHQ = Patient Health Questionnaire-9, SWLS = Satisfaction With Life Scale, ULS = UCLA Loneliness Scale.

The NCT revealed no significant differences between the male and female networks in the global network structure (network invariance test: *M* = 0.20, *p* = .78) as well as connectivity (global strength invariance test: *S* = 0.42, *p* = .19; *S*_males_ = 11.24, *S*_females_ = 10.83). However, the strength values of “restlessness” (GAD5) and “tiredness” (PHQ4) were significantly higher in the male network than in the female network (*p* < .05). In the male network, nodes with higher centrality included “trouble relaxing” (GAD4), “tiredness” (PHQ4), “feeling isolated from others” (ULS3), and “ideal life” (SWLS1) (Fig. 4, Table S3). Conversely, in the female network, the central nodes were “uncontrollable worry” (GAD2), “sad mood” (PHQ2), “feeling isolated from others” (ULS3), and “life in excellent condition” (SWLS2) (Fig. 4, Table S3). Centrality values for each node are detailed in Table S3. There were significant differences in the 16 edge weights between the male and female networks (Table 2).

**Table 2:** Significant differences in edge weights between the male and female networks by network comparison test.

Edge		E-value
GAD4	ULS1	0.004*
GAD4	SWLS3	0.076*
GAD5	PHQ5	0.136*
GAD5	PHQ8	0.193*
GAD5	SWLS2	0.041*
GAD5	SWLS3	0.015*
GAD7	PHQ9	0.190*
GAD7	ULS3	0.063*
PHQ3	PHQ5	0.203*
PHQ4	SWLS4	0.056*
PHQ5	ULS2	0.072*
PHQ5	SWLS3	0.044*
PHQ7	SWLS3	0.001*
PHQ9	SWLS4	0.069*
ULS1	SWLS5	0.075*
SWLS4	SWLS5	0.160*

*p < 0.05.

GAD generalized anxiety disorder-7, *PHQ* patient health questionnaire-9, *SWLS* satisfaction with life scale, *ULS* UCLA loneliness scale.

## Discussion

The present study examined the network structure of mental health consisting of symptoms of depression and anxiety, loneliness, and satisfaction with life in the Japanese population. In the overall network, central symptoms were “trouble relaxing (symptom of anxiety)”, “sad mood (symptom of depression)”, “feeling isolated from others (loneliness)”, and “life in excellent condition (satisfaction with life)”. The centrality of “sad mood” aligns with findings from previous studies^13–15^. Thus, feelings of depression may be a central symptom of mental health across cultures. However, unlike these previous studies, the strength value for “trouble relaxing” was notably high in this Japanese sample, and it was strongly associated with “sad mood” even after controlling for all other nodes. Moreover, the trouble relaxing node showed associations with all other symptoms of anxiety. Therefore, problems relaxing may also play an important role in the development of poor mental health in the Japanese population.

Interestingly, the interconnections between depression, loneliness, and life satisfaction, as well as between anxiety, loneliness, and life satisfaction, were not very high. Similarly, Yang et al.^17^ showed low associations between nodes of loneliness and anxiety (weight = 0.00 to 0.07) or symptoms of depression (weight = 0.00 to 0.06). This suggests that each symptom of anxiety and depression has a low effect on loneliness and life satisfaction. While loneliness demonstrated strong internal associations among its variables, its connections with life satisfaction were very weak. Similarly, life satisfaction showed strong internal associations within its variables. Therefore, loneliness and life satisfaction may represent independent clusters.

Our study found that females exhibited significantly higher depressive symptoms related to sleep difficulties, appetite, and concentration problems compared to males. However, no significant differences were observed in network structure and connectivity between the two groups, suggesting that males and females have similar psychopathology in mental health. Nevertheless, specific differences emerged. The strength values of “restlessness” and “tiredness” were significantly higher in the male network than in the female network. Furthermore, “restlessness” was strongly associated with “motor” in the male network, but “restlessness” was not associated with “motor” in the female network. This result suggests that excessive restlessness (inability to sit still) in males may contribute to psychomotor decline (moving or speaking either much more slowly or significantly faster than usual). Conversely, “tiredness” showed a slight negative association with “accomplishment” in the male network but not in the network for females. This suggests that, among males, excessive tiredness (feeling tired or lacking energy) may be linked to a diminished sense of accomplishment (the feeling of having something important in life). As a result, restlessness may play a key role in exacerbating depression and anxiety, while tiredness may contribute to worsening depressive symptoms and lower life satisfaction in males. Regarding the association between mental health problems and life satisfaction in the female network, “anhedonia” showed a relatively strong negative association with “ideal life” (weight = -0.123). This finding suggests that anhedonia and the perception of living an ideal life may play a crucial role in exacerbating depressive symptoms and lower life satisfaction in females.

Our findings indicate that depression and anxiety are closely interrelated, whereas loneliness and life satisfaction appear to form independent clusters with less direct association with depression and anxiety. Furthermore, problems relaxing and feelings of depression may contribute significantly to the development of depression and anxiety, while feelings of isolation may underlie the increase in loneliness. Conversely, experiencing life in excellent condition may improve life satisfaction. These findings suggest that these components could serve as potential intervention targets to improve mental health within the Japanese population.

A possible intervention for problems relaxing may be mindfulness and relaxation practices. Relaxation has been shown to be effective not only for reducing anxiety, but also depression^31^. Similarly, mindfulness-based practices have been found to reduce symptoms of anxiety and depression^32^. Another intervention to address depression, as well as feelings of isolation and life satisfaction may be behavioral activation^33^. It may be beneficial to increase constructive behavior patterns by creating an activity recording schedule to help them live in excellent condition, lower their sad moods, and lower their isolation from others. Previous studies have shown that behavioral activation effectively reduces depressive symptoms and isolation while improving well-being^34–36^. Although the number of suicides in Japan is relatively high^3^, the present network showed a strong association between “suicidal ideation”, “worthlessness”, and “motor”. This result aligns with findings by Yang et al.^17^; however, unlike the previous study, the present study observed a stronger association between feelings of worthlessness and suicidal ideation than between suicidal ideation and psychomotor. This finding suggests that addressing thoughts of worthlessness through cognitive restructuring could potentially prevent suicidal ideation for the Japanese. Based on the above, providing cognitive-behavioral therapy including relaxation, behavioral activation, and cognitive restructuring might prevent mental problems among the Japanese general population from worsening. Future studies are needed to confirm the effectiveness of these interventions.

However, this study had several limitations. First, the interpretation of betweenness was not feasible in this analysis because its CS values were below 0.25. While strength is regarded as the most crucial index in psychometric networks^12^, closeness and betweenness capture distinct connectivity aspects. Consequently, a larger sample size is required to re-evaluating these findings. Second, the study employed a cross-sectional design. Given that the edges represented partial correlation coefficients without directionality, causal inferences among them could not be drawn. To address this, future research should utilize longitudinal network analysis to explore causal relationships and monitor temporal dynamics. Third, the network examined in this study included only symptoms of depression and anxiety, loneliness, and life satisfaction. These variables do not encompass all components of mental health. It is necessary to explore the network structure of mental health, incorporating additional factors. Fourth, this study did not take socio-demographic factors into account. Since these factors influence mental health^20^, future research should conduct a moderated network analysis using socio-demographic factors as moderator variables. Fifth, this study grouped participants by biological sex. Future research should aim to compare network structures that account for LGBTQ + identities, as stress associated with these identities significantly influences mental health and represents a crucial aspect of its diversity^37^. Given that network structures may vary across cultures^38^, cross-cultural comparisons should also be conducted in future studies.

## Conclusions

Our study aimed to explore the network structure of mental health, encompassing symptoms of anxiety and depression, loneliness, and satisfaction with life. The findings revealed no significant differences in the global network structure and connectivity of mental health symptoms between males and females. Within the overall network, central symptoms were identified as “trouble relaxing,” “sad mood,” “feeling isolated from others,” and “life in excellent condition”. These symptoms may play pivotal roles in the development of poor mental health among the Japanese population and represent potential targets for intervention. Addressing these central symptoms could contribute to the improvement of mental health outcomes in Japan. This study underscores the need for further research into the network structure of mental health in the Japanese context. Future studies should build upon these findings while addressing the limitations of the present study, including the need for longitudinal designs. Rigorous research is essential to advance clinical applications and to develop effective interventions for mental health challenges in Japan.

## Electronic supplementary material

Below is the link to the electronic supplementary material.

Supplementary Material 1

## Data Availability

Detailed data are available from the corresponding authors upon reasonable request.

## Abbreviations

CS: Correlation stability
GAD: Generalized anxiety disorder-7
NCT: Network comparison test
PHQ: Patient health questionnaire-9
SWLS: Satisfaction with life scale
ULS: UCLA loneliness scale
