# Supplementary material for: Network structure of common mental health problems and life satisfaction in a Japanese population
Source: Sci Rep. 2025 Apr 10;15:12325. doi: 10.1038/s41598-025-95554-1 (PMC11986124; doi:10.1038/s41598-025-95554-1)
Supplement: Supplementary file 1 — Supplementary Material 1 [file 41598_2025_95554_MOESM1_ESM.docx]

Supplemental material

**Table S1** *Socio-demographic data*

**Table S2** *Edge weights in the network for all participants*

*Note.* GAD = Generalized Anxiety Disorder-7, PHQ = Patient Health Questionnaire-9, SWLS = Satisfaction With Life Scale, ULS = UCLA Loneliness Scale

**Table S3** *Strength, closeness, and betweenness centrality values for each node*

*Note.* GAD = Generalized Anxiety Disorder-7, PHQ = Patient Health Questionnaire-9, SWLS = Satisfaction With Life Scale, ULS = UCLA Loneliness Scale

**Table S4** *Edge weights in the network for males*

*Note.* GAD = Generalized Anxiety Disorder-7, PHQ = Patient Health Questionnaire-9, SWLS = Satisfaction With Life Scale, ULS = UCLA Loneliness Scale

**Table S5** *Edge weights in the network for females*

*Note.* GAD = Generalized Anxiety Disorder-7, PHQ = Patient Health Questionnaire-9, SWLS = Satisfaction With Life Scale, ULS = UCLA Loneliness Scale

**Figure S1** *Accuracy of edge weights for all participants (N = 476)*.


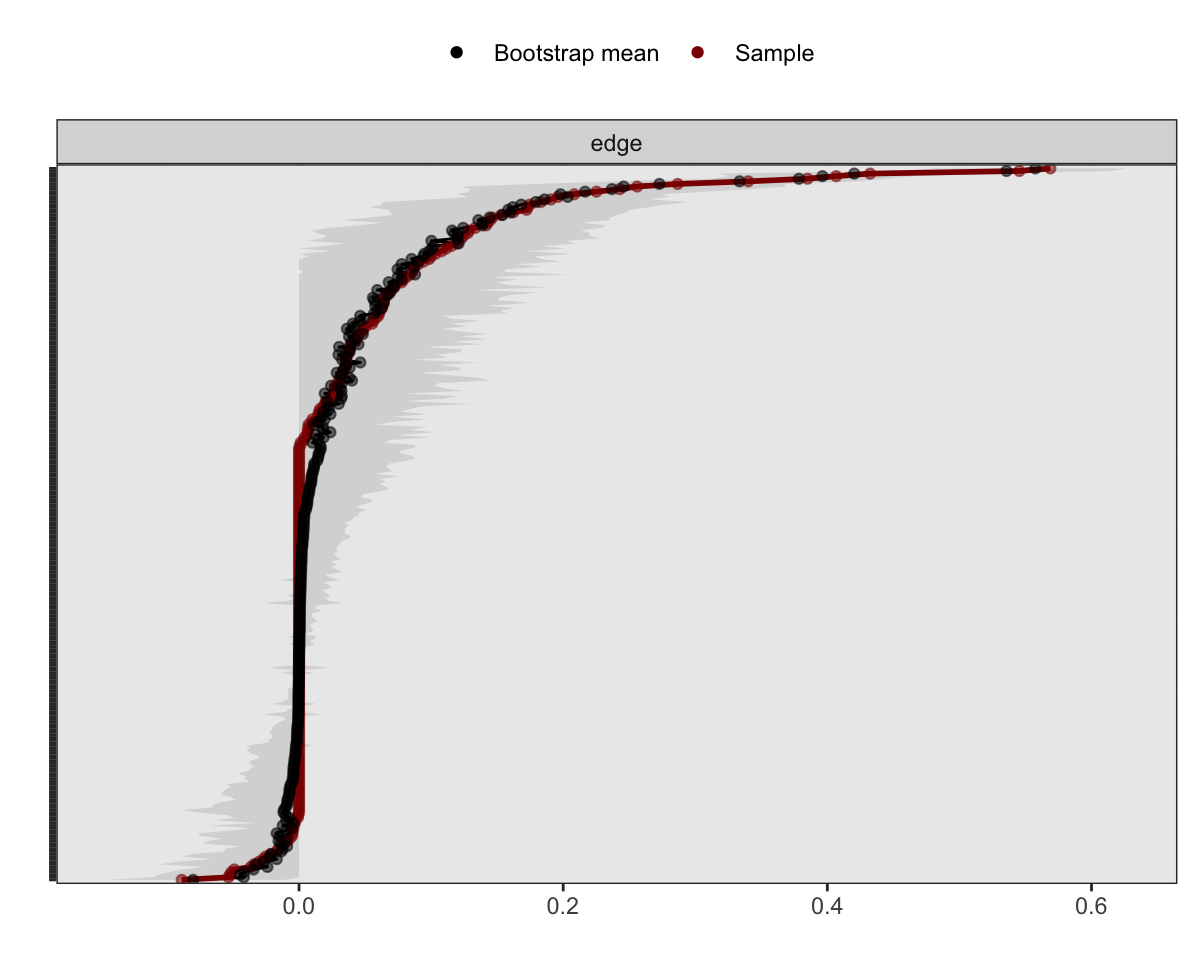


Note. The red lines indicate the edge values estimated from the samples, while the black lines represent the average bootstrapped edge values. The gray area shows the 95% confidence intervals obtained through bootstrapping. Each horizontal line corresponds to an edge, ordered by edge weight from highest to lowest.

**Figure S2** *Stability of betweenneess, closeness, and strength centrality for all participants (N = 476)*.


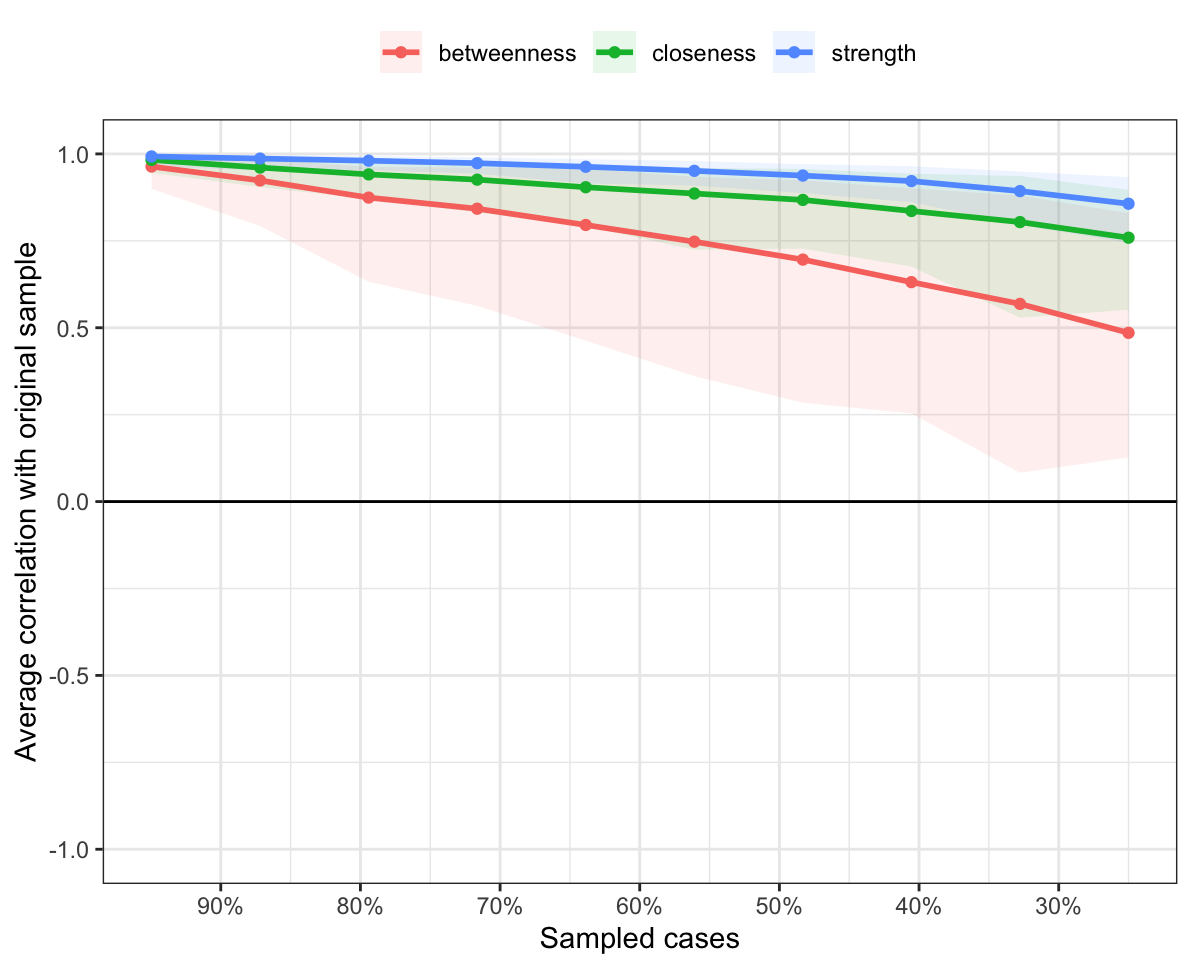


Note. The lines depict the mean correlation between centrality indices from the original subsample and those derived from the case-dropping bootstrap. The shaded area around the lines indicates the range of correlations, spanning from the 2.5th to the 97.5th quantile.

**Figure S3** *Accuracy of edge weights for males (n = 235) and females (n = 241).*

*
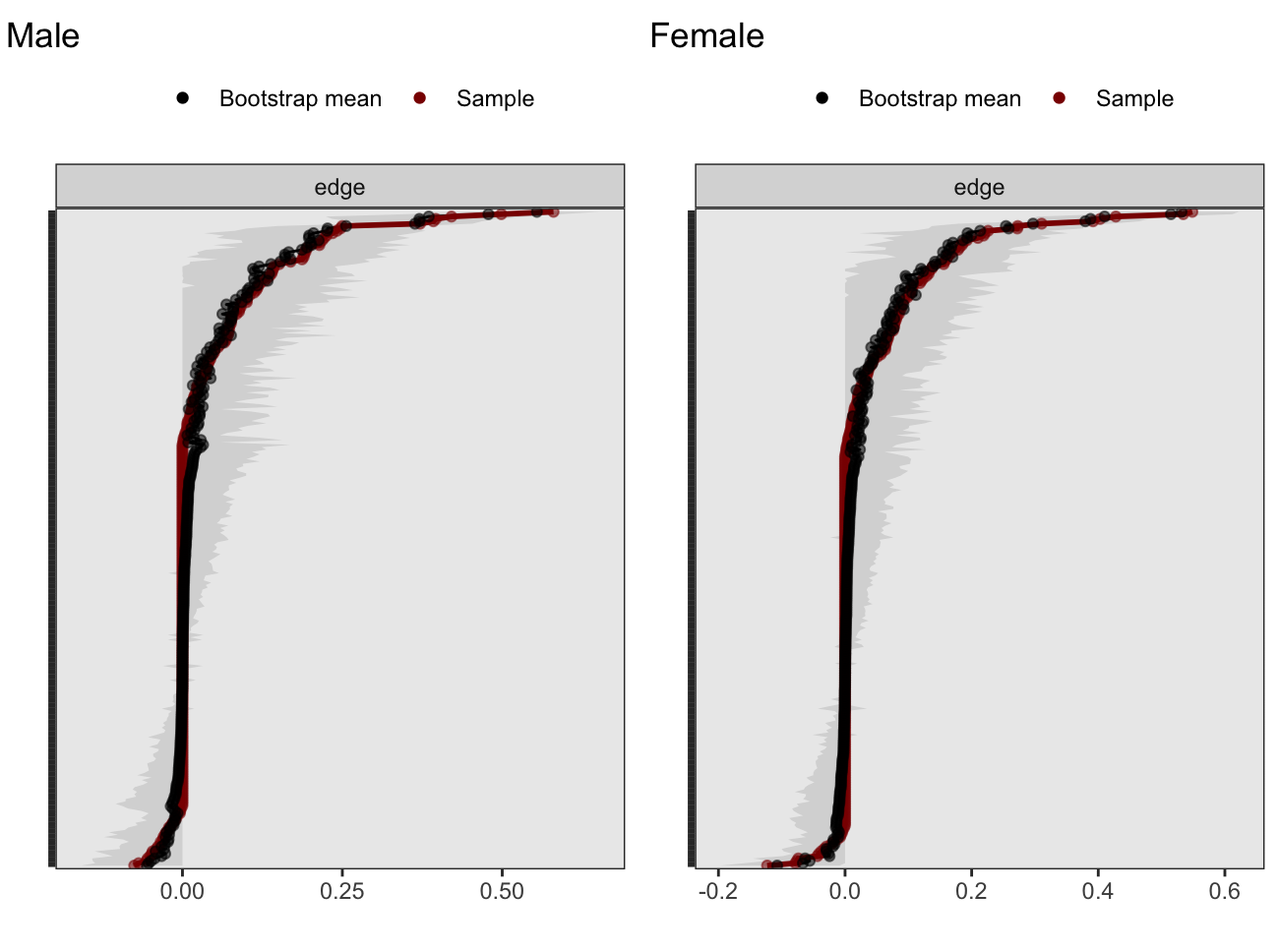
*

Note. The red lines indicate the edge values estimated from the samples, while the black lines represent the average bootstrapped edge values. The gray area shows the 95% confidence intervals obtained through bootstrapping. Each horizontal line corresponds to an edge, ordered by edge weight from highest to lowest.

**Figure S4** *Stability of betweenneess, closeness, and strength centrality for males (n = 235) and females (n = 241).*

*
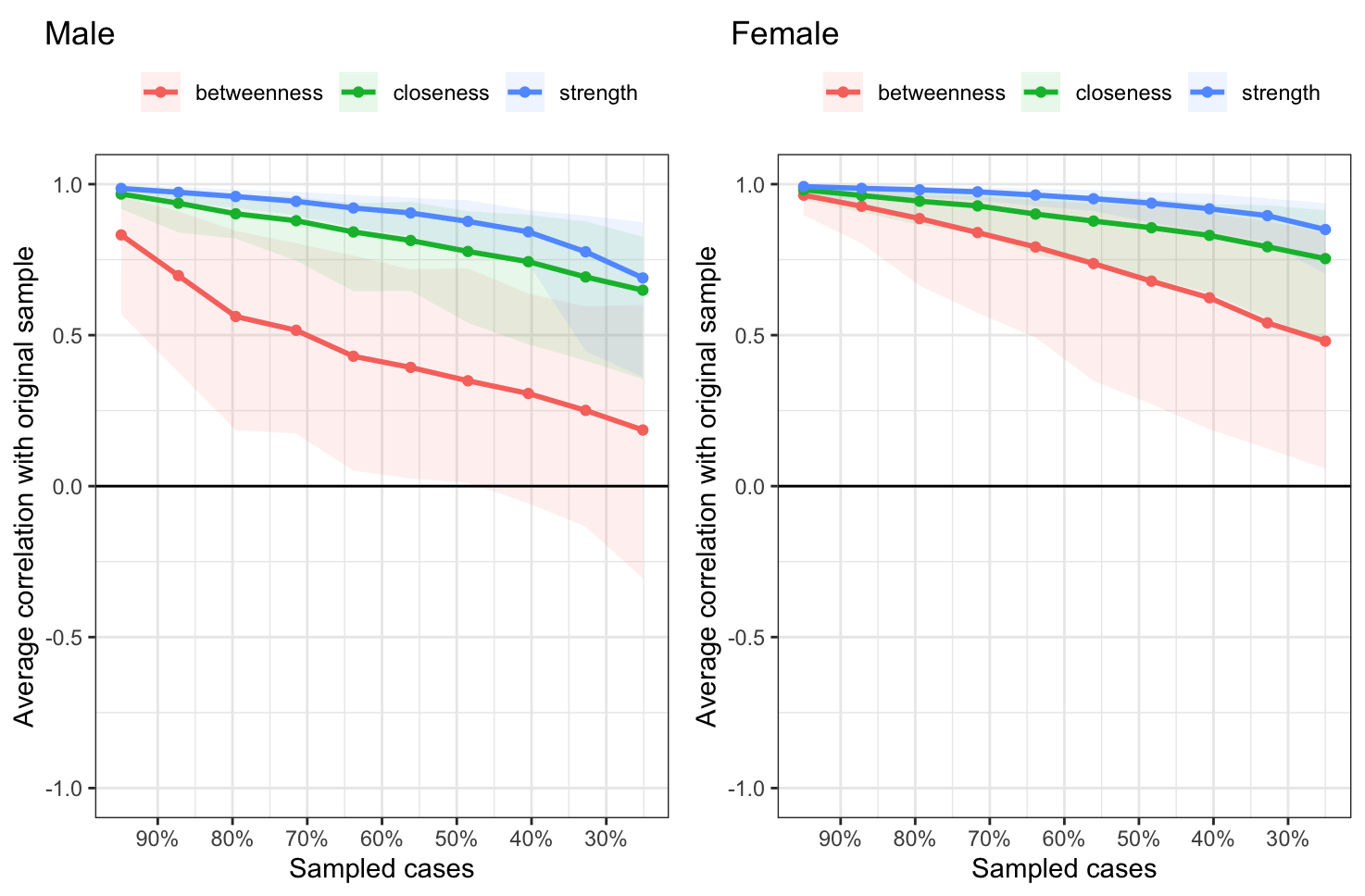
*

Note. The lines depict the mean correlation between centrality indices from the original subsample and those derived from the case-dropping bootstrap. The shaded area around the lines indicates the range of correlations, spanning from the 2.5th to the 97.5th quantile.
